# Role of Pathogenicity Determinant Protein C (PdpC) in Determining the Virulence of the *Francisella tularensis* Subspecies *tularensis* SCHU

**DOI:** 10.1371/journal.pone.0089075

**Published:** 2014-02-18

**Authors:** Akihiko Uda, Tsuyoshi Sekizuka, Kiyoshi Tanabayashi, Osamu Fujita, Makoto Kuroda, Akitoyo Hotta, Naoko Sugiura, Neekun Sharma, Shigeru Morikawa, Akio Yamada

**Affiliations:** 1 Department of Veterinary Science, National Institute of Infectious Diseases, Shinjuku, Tokyo, Japan; 2 Pathogen Genomics Center, National Institute of Infectious Diseases, Shinjuku, Tokyo, Japan; 3 United Graduate School of Veterinary Science, Gifu University, Gifu, Japan; 4 Laboratory of Veterinary Public Health, Graduate School of Agricultural and Life Science, The University of Tokyo, Bunkyo, Tokyo, Japan; University of Louisville, United States of America

## Abstract

*Francisella tularensis* subspecies *tularensis*, the etiological agent of tularemia, is highly pathogenic to humans and animals. However, the SCHU strain of *F. tularensis* SCHU P0 maintained by passaging in artificial media has been found to be attenuated. To better understand the molecular mechanisms behind the pathogenicity of *F. tularensis* SCHU, we attempted to isolate virulent bacteria by serial passages in mice. SCHU P5 obtained after 5th passages in mice remained avirulent, while SCHU P9 obtained after 9th passages was completely virulent in mice. Moreover, SCHU P9 grew more efficiently in J774.1 murine macrophages compared with that in the less pathogenic SCHU P0 and P5. Comparison of the nucleotide sequences of the whole genomes of SCHU P0, P5, and P9 revealed only 1 nucleotide difference among P0, P5 and P9 in 1 of the 2 copies of pathogenicity determinant protein C (*pdpC*) gene. An adenine residue deletion was observed in the *pdpC1* gene of SCHU P0, P5, and P9 and in the *pdpC2* gene of SCHU P0, and P5, while P9 was characterized by the wild type *pdpC2* gene. Thus, SCHU P0 and P5 expressed only truncated forms of PdpC protein, while SCHU P9 expressed both wild type and truncated versions. To validate the pathogenicity of PdpC, both copies of the *pdpC* gene in SCHU P9 have been inactivated by Targetron mutagenesis. SCHU P9 mutants with inactivated *pdpC* gene showed low intracellular growth in J774.1 cells and did not induce severe disease in experimentally infected mice, while virulence of the mutants was restored by complementation with expression of the intact PdpC. These results demonstrate that PdpC is crucial in determining the virulence of *F. tularensis* SCHU.

## Introduction

*Francisella tularensis*, the etiological agent of tularemia, is a gram-negative, facultative intracellular bacterium. Tularemia can be transmitted by insect bites, inhalation of contaminated aerosols, and ingestion of contaminated food or water [1], [2]. Human-to-human transmission has not been reported [3]. Due to its high pathogenicity and infectiousness, this bacterium is listed as a category A bioterrorism agent by the Centers for Disease Control and Prevention [3], [4].

Three known subspecies (subsp.) of *F. tularensis* are *tularensis*, *holarctica*, and *mediasiatica*. The SCHU strain of *F. tularensis* subsp. *tularensis*, isolated from a patient in the United States of America in 1941 [5], [6], has been shown to be highly pathogenic in experimentally infected mice. The 50% lethal dose (LD_50_) for intraperitoneally inoculated mice is <10 colony forming units (CFU), and death occurs within a week after inoculation [4], [7], [8]. However, extensive passage of the SCHU strain on artificial media resulted in the appearance of low-pathogenic mutants such as FSC043 [9], [10].

*Francisella* can use several receptors to enter macrophages, including complement receptor 3 (CR3) [11], [12]. However, *F. tularensis* escapes from the phagosome of the macrophages, migrates into the cytosol, and replicates [13]. Phagosomal escape is facilitated by several bacterial genes residing on the *Francisella* pathogenicity island (FPI), which are duplicated in the *F. tularensis* SCHU genome [14], [15]. Sixteen to nineteen genes located in the 33-kb FPI are induced during growth in macrophages [16]–[20], most of which are indispensable for *Francisella* growth in macrophages as well as for virulence in mice or flies [21]. However, two controversial reports on the contribution of *pdpC* to virulence were published. The *pdpC* mutant produced by transposon insertion from the *Francisella novicida* U112 strain exhibits virulence in the fruit fly and the mosquito Sua1B cell line compared with that of wild-type bacteria [22], [23]. In contrast, *pdpC* mutants created from the *F. tularensis* subsp. *tularensis* Schu S4 and subsp. *holarctica* LVS strains showed defective replication in primary human monocyte-derived macrophages, J774.1 murine macrophages, and mice [24], [25]. Therefore, the contribution of PdpC protein toward virulence of *F. tularensis* is still unclear. In this study, we found that SCHU strain (SCHU P0) maintained by passaging in artificial media was attenuated as for FSC043 strain and virulence of SCHU P0 was rescued after 9th passages (SCHU P9) in mice. We found a frameshift mutation within *pdpC* in FPI of SCHU P0 resulting in truncation of PdpC protein, which was recovered by a single adenine insertion in SCHU P9. To clarify the function of PdpC protein, *pdpC* knockout mutations in SCHU P9 and *pdpC*-complemented SCHU strains were analyzed for their intracellular growth and virulence in mice.

## Materials and Methods

### Ethics Statement

This study protocol was submitted for the NIID biosafety committee review and the committee approved the protocol did not correspond to a dual use research of concern (Permission Number:62, 62-2, 62-3, 75, 75-2, and 75-3). This study was performed in strict accordance with the Fundamental Guidelines for Proper Conduct of Animal Experiment and Related Activities in Institutions under the jurisdiction of the Ministry of Health, Labour and Welfare, Japan (June 2006). The protocol was approved by the Animal Care and Use Committee of NIID (Permission Number: 209070 and 212003).

### Bacteria

The SCHU P0 strain of *F. tularensis* subsp. *tularensis* (SCHU P0) was kindly provided by Dr. H. Fujita (Ohara Research Laboratory, Ohara General Hospital, Fukushima, Japan). Its precise passage history is unknown. On arrival, bacteria were cultured on chocolate II agar (Becton Dickinson, Sparks, MD, USA) at 37°C for 3 days, resuspended in saline containing 10% glycerol, and stored at −80°C until use. All work with live bacterial cultures was performed in a biosafety level 3 facility, in accordance with the regulations stipulated by the National Institute of Infectious Diseases (NIID), Japan.

### Serial Passages of *F. tularensis* in Mice

**Figure 1:**
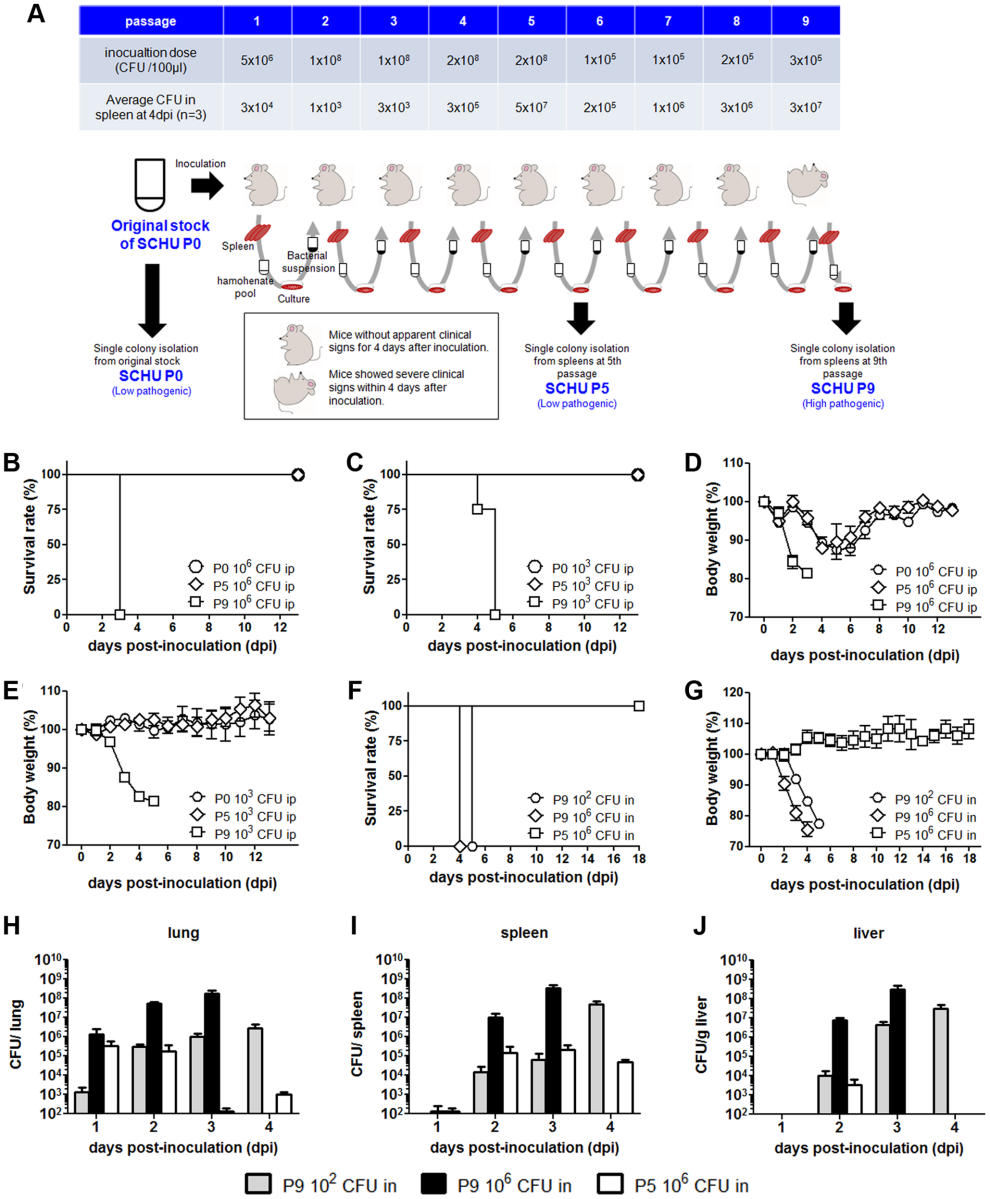
Pathogenicity of *Francisella* strains in mice. (A) The scheme for SCHU P0, P5, and P9 isolation. Three C57BL/6J mice were inoculated intraperitoneally with 5×10^6^ CFU ml^−1^ of SCHU P0 suspension and mice were sacrificed 4 days after infection. Three mice spleens obtained from the infected mice were homogenized. One hundred micro litter of 10% spleen homogenate was plated on chocolate II agar, and cultured for 3 days. After cultivation, the bacteria grown confluently on the plate were suspended with 1 ml of chemically defined medium (CDM). Subsequent passages were performed similarly. When mice demonstrated severe clinical signs or >20% weight loss, they were humanely sacrificed by isoflurane inhalation. If the mice did not show apparent clinical signs, they were sacrificed 4 days after infection. The inoculation dose and CFU in spleen at 4 days post-inoculation in each passage were shown in top table. Spleen homogenates prepared at the 5th and 9th passages were cultured on chocolate II agar. Single colonies were then isolated and designated as SCHU P5 and P9, respectively. Bacterial stocks were prepared by cultivating respective strains in CDM at 37°C for 24 h and stored in CDM containing 10% glycerol at −80°C until use. (B–E) Four C57BL/6J mice from each group were intraperitoneally infected with 1×10^6^ (B, D) or 1×10^3^ (C, E) CFU of the SCHU P0 (circle), P5 (diamond), or P9 (square) strains. Their survival rates (B, C) and body weights (D, E) were measured and observed for 14 days. Survival rates and mean ± standard error of the mean (SEM) of percentage body weights are shown in the upper and lower panels, respectively. (F–J) Three C57BL/6J mice in each group were intranasally inoculated with 10^2^ CFU SCHU P9 (circle), 10^6^ CFU SCHU P9 (diamond), and 10^6^ CFU SCHU P5 (square). Their survival rates (F) and body weights (G) were measured and observed for 18 days. Mice were sacrificed at the indicated days post-inoculation, and then the averages ± SEM of bacterial CFU in lungs (H), spleens (I), and livers (J) were shown. The data in CFU of SCHU P9 10^6^ CFU at 4 dpi were defected because all the mice were sacrificed within 3 dpi.

Figure 1A illustrates the scheme for SCHU P0, P5, and P9 isolation. SCHU P0 was cultured to confluence for 3 days on chocolate II agar (Becton Dickinson and Co., Cockeysville, MD) from its glycerol stock and resuspended in saline. Three C57BL/6J mice were inoculated intraperitoneally with 5×10^6^ CFU ml^−1^ of SCHU suspension and mice were sacrificed 4 days after infection. Three spleens obtained from the infected mice were homogenized. One hundred microlitter of 10% spleen homogenate was plated on chocolate II agar, and cultured for 3 days. After cultivation, the bacteria grown confluently on the plate were suspended with 1 ml of chemically defined medium (CDM). Subsequent passages were performed similarly. When mice demonstrated severe clinical signs or >20% weight loss, they were humanely sacrificed by isoflurane inhalation. If the mice did not show apparent clinical signs, they were sacrificed 4 days after infection. The inoculation dose and CFU in spleen at 4 days post-inoculation in each passage are shown in figure 1A. Spleen homogenates prepared at the 5th and 9th passages were cultured on chocolate II agar. Single colonies were then isolated and designated as SCHU P5 and P9, respectively. Bacterial stocks were prepared by cultivating respective strains in CDM at 37°C for 24 h and stored in CDM containing 10% glycerol at −80°C until use.

### Short-read DNA Sequencing Using the Illumina Genome Analyzer II

Mixtures of bacterial genomic DNA were extracted using SepaGene (Sanko Junyaku, Tokyo, Japan) according to the manufacturer’s instructions. After samples were treated with 100 µg ml^−1^ ribonuclease (DNase-free) solution (Nippon Gene, Tokyo, Japan) in TE buffer (Nippon Gene, pH 8.0) at 37°C for 30 min and stored at 4°C for 3 days, the genomic DNA was extracted using phenol/chloroform method and recovered by ethanol precipitation.

A DNA library of approximately 600-bp was prepared using a genomic DNA sample prep kit (Illumina, San Diego, CA, USA), and DNA clusters were generated on a slide using a cluster generation kit (ver. 2) in the Illumina cluster station according to the manufacturer’s instructions. To obtain approximately 1×10^7^ clusters per lane, the general procedure as described in the standard recipe was employed in the following order: template hybridization, isothermal amplification, linearization, blocking, denaturation, and hybridization of the sequencing primer (Illumina). All sequencing runs for 80-mers were performed with an Illumina Genome Analyzer II (GA II) using the Illumina sequencing kit (ver. 3). Fluorescent images were analyzed using the Illumina base-calling pipeline 1.5.0 to obtain FASTQ-formatted sequence data.

### Read Mapping

To extract single nucleotide variations (SNV) and insertion–deletion (Indel) sites in the sequences of the SCHU P0, P5, and P9 strains, the 40-mer forward and reverse reads constructed from 80-mer reads were aligned to SCHU S4 (NC_006570) as a reference sequence with Maq software (ver. 0.7.1) [26] using the easyrun Perl command. Division into 40-mer reads is effective for identifying Indel sites in addition to SNV. The SNV and Indel sites were extracted as “cns. final. snp” and “cns. indelpe” files.

### Short-read Archive Accession Numbers

Short read archives have been deposited in the DNA Data Bank of Japan (DDBJ accession number: DRA000207).

### PCR and Nucleotide Sequencing

Primer pairs targeting *pdpC* were designed using DNASIS Pro (Hitachi Software, Tokyo, Japan) and synthesized by Hokkaido System Science (Hokkaido, Japan). Primer pairs used in this study are shown in Table S1. PCR was performed with a GeneAmp PCR System 9700 (Perkin Elmer, Foster City, CA, USA) in a 20-µl reaction mixture containing 1 U Takara *ExTaq* (Takara, Shiga, Japan), 1× PCR buffer supplied with enzyme, 0.5 µM primers, 250 µM of each dNTP, and 1 ng bacterial genomic DNA. Reaction conditions were as follows: initial denaturation at 94°C for 5 min, 30 cycles of 94°C for 0.5 min, 55°C for 0.5 min, and 72°C for 0.5 min and final extension at 72°C for 7 min. Amplicons were purified and sequenced using the BigDye version 3.1 terminator cycle sequencing kits (Applied Biosystems, Foster City, CA, USA) and the 3130 DNA sequencer (Applied Biosystems) according to the manufacturer’s instructions. PI1F/PI1R and PI2F/PI2R primer pairs shown in Table S1 were designed to independently amplify the duplicated FPI in *F. tularensis* SCHU. The long DNA fragments (approximately 36.5-kbp) encompassing the entire FPI, together with the flanking regions, were amplified using the long PCR method. The composition of the reaction mixture was similar to that described above, except that 1 U Takara *LATaq* (Takara) was used. Reaction conditions were as follows: an initial denaturation at 94°C for 1 min, 30 cycles of 98°C for 10 s, 68°C for 15 min, and final extension at 72°C for 10 min.

### Western Blotting

Bacteria grown in CDM and harvested at late log phase (OD_600_ = 2.0) were solubilized for 15 min at 94°C with 2× Laemmli sample buffer (Biorad, Hercules, CA). The samples were loaded onto a 12.5% acrylamide gel and electrophoresis was performed at 20 mA for 1.5 h. The gel was soaked in transfer buffer (12.5 mM Tris, 100 mM glycine, and 10% methanol) for 10 min and proteins were electroblotted onto PVDF membranes at 0.8 mA cm^−2^ for 1.5 h. The membrane was blocked with Blocking One (Nacalai Tesque, Kyoto, Japan) for 1 h and washed twice with PBS containing 0.1% Tween 20 (PBS-T). The membranes were incubated with rabbit polyclonal anti-PdpC antibodies, a generous gift from Dr. J. Celli (NIH, NIAID, Hamilton, MT), at a dilution of 1∶20,000 in dilution buffer (1∶10 dilution of Blocking One) [20]. Similarly, rabbit polyclonal anti-GapA antibodies raised against the synthetic peptide CSEDASGFSPPEDSFS were used at a dilution of 1∶20,000 in dilution buffer as a loading control. After the membrane was washed thrice with PBS-T, it was incubated with protein A-horseradish peroxidase conjugate at a dilution of 1∶20,000 in dilution buffer (BioRad, Richmond, CA, USA). After three washes with PBS-T, reactions were visualized with ECL Plus or ECL prime reagents (GE Healthcare, Piscataway, NJ, USA). The images were captured with a VersaDoc 5000 imaging system (Bio-Rad).

### Plasmid Construction for Generating *F. tularensis* pdpC Mutant

The *F. tularensis pdpC* mutant was generated by group II intron insertion using the TargeTron gene knockout system (Sigma-Aldrich) and pKEK1140 plasmid (GenBank accession number: EU499313), which was a kind gift from Dr. Karl E. Klose (South Texas Center for Emerging Infectious Diseases and Department of Biology, University of Texas at San Antonio) [27]. The IBS, EBS1d, and EBS2 primer sets used for specific insertion at nucleotide positions 538, 1119, and 2013 of *pdpC* were designed by the Sigma-Aldrich computer-based TargeTron algorithm (http://www. sigma-genosys. com/targetron/). However, the *Hind*III restriction site on the IBS primer was replaced by a *Xho*I restriction site for cloning into pKEK1140. These primers, shown in Table S1, were synthesized by Operon Technology (Tokyo, Japan). The retargeted PCR products (350-bp) were generated using splicing by overlap extension PCR with IBS, EBS1d, EBS2, and EBS universal primers, according to the manufacturer’s instructions. The PCR product treated with *Xho*I (New England Biolabs, Beverly, MA, USA) and *Bsr*GI (New England Biolabs) restriction enzymes was ligated into pKEK1140 digested with the same enzymes. After amplification in transformed *Escherichia coli* DH5α (Competent high DH5α, Toyobo, Tokyo, Japan) the plasmid was purified using a NucleoBond PC 100 (Macherey-Nagel GmbH &Co., Doren, Germany). These plasmids were designated as pKEK(pdpC538 ins), pKEK(pdpC1119 ins), and pKEK(pdpC2038 ins).

### Generation of Knockout Mutants of pdpC in SCHU P9

Virulent *F. tularensis* SCHU P9 was cultured overnight with vigorous aeration at 37°C until the cell density reached approximately OD_600_ = 0.7. Bacterial cells harvested by centrifugation at 12,000×*g* for 2 min were washed thrice with 0.5 M sucrose and resuspended in 0.5 M sucrose. Two micrograms each of pKEK(pdpC538 ins), pKEK(pdpC1119 ins), and pKEK(pdpC2013 ins) were electroporated into the cells using a Bio-Rad micropulser (Bio-Rad) at 2.5 kV. The transformed bacteria were precultured in CDM at 30°C for 1.5 h, and cultured on chocolate Eugon agar plates (Becton Dickinson) containing 50 µg ml^−1^ kanamycin at 30°C. After additional plate streaking, mutagenesis was confirmed using PCR, detecting 915-bp insertions at nucleotide positions 538, 1119, and 2013 of *pdpC* using the pdpC-435F/pdpC-2240R primer pair (Table S1). To remove the bacterial intracellular plasmids, the *F. tularensis pdpC* mutants were further cultured on chocolate Eugon agar plates (Becton Dickinson) without antibiotics at 37°C. Loss of plasmid was confirmed by the lack of growth on CDM containing kanamycin. The resultant *pdpC* knockout mutants were referred to as 538 ins, 1119 ins, and 2013 ins mutants.

### Confirmation of the Inserted Sequence in the pdpC Mutants

Genomic DNA was extracted from SCHU P9, 538 ins, 1119 ins, and 2013 ins mutants using SepaGene (Sanko Junyaku) according to the manufacturer’s instructions. PCR was performed by KOD FX Neo (Toyobo) using primer pairs (pdpC-435F/pdpC-2240R, pdpC-435F/EBS universal, and EBS universal/pdpC-2240R; Table S1). The reaction mixtures were denatured at 94°C for 2 min, followed by 30 cycles of 94°C for 0.5 min, 55°C for 0.5 min, and 72°C for 0.5 min. The amplicons and smart ladder (0.2–10 kbp; Nippongene, Tokyo, Japan) were electrophoresed on a 0.7% agarose gel.

### Complementation of Avirulent Phenotype of the Bacteria with Wild Type pdpC

To complement the avirulent and knockout strains with PdpC, we constructed the pNVU1 expression plasmid, composed of three DNA fragments: the *Francisella bfr* promoter [28], the tetracycline resistance gene, and a partial pOM5 plasmid [29]. *Francsiella bfr* promoter was amplified by PCR from SCHU P9 genomic DNA using Pbfr-s/Pbfr-a primers (Table S1). The tetracycline resistance gene from the pBR322 plasmid and a partial pOM5 plasmid were amplified using pNVU1-s/pNVU1-a and tet-s/tet-a primer pairs, respectively (Table S1). Three PCR-generated DNA fragments containing 15-bp overlap each other were connected using an In-Fusion HD Cloning Kit (Clontech Laboratories, Mountain View, CA). In pA9 and pA8 plasmids, the tetracycline resistance gene on pNVU1 plasmid was replaced with *pdpC* containing either an 8-A or 9-A stretch at nucleotide position 2037, which were amplified from SCHU P9 genomic DNA using a pdpC-s/pdpC-a primer pair (Table S1). pA9 and pA8 plasmids were replicated in *E. coli* DH5α and purified using NucleoBond PC 100 (MACHEREY-NAGEL GmbH & Co. ). A virulent SCHU P5 and *pdpC* knockout 538 ins mutants were transformed with these plasmids by electroporation. The transformed bacteria were precultured in CDM at 37°C for 1 h, and then selected on chocolate Eugon agar plates (Becton Dickinson) containing 7.5 µg ml^−1^ chloramphenicol at 37°C for 3 days. Bacterial stocks were prepared by cultivating respective strains in CDM at 37°C for 24 h and stored in CDM containing 10% glycerol at −80°C until use.

### In vitro Intracellular Bacterial Growth in Phagocytes

J774.1 murine macrophages, obtained from the RIKEN cell bank (RIKEN Bioresource Center, Ibaraki, Japan), were used for assessing intracellular bacterial growth in phagocytes. All experiments were performed in triplicate. J774.1 cells stimulated with 1 ng ml^−1^ phorbol-12-myristate 13-acetate (PMA, Sigma-Aldrich, St. Louis, MO) in RPMI1640 (Wako, Osaka, Japan) containing 100 U ml^−1^ penicillin and 10% fetal bovine serum (FBS, Invitrogen, Auckland, New Zealand) at 37°C for 2 days, were further cultured in the medium without PMA for 2 days. Cells prepared in wells of a Multiwell Primaria 24 well (Falcon, Franklin Lakes, NJ, USA) were inoculated with approximately 1×10^6^ CFU of the bacteria, which is equivalent to a multiplicity of infection (MOI ) of 10. After centrifugation at 1000×*g* for 10 min, the cells were incubated for 1 h. Cells were then washed thrice with RPMI1640 medium and were cultured in RPMI1640 containing 100 U ml^−1^ penicillin, 50 µg ml^−1^ gentamicin, and 10% FBS. After the incubation period, the cells were washed thrice with PBS, treated with 100 µl 0.1% Triton X-100 in CDM for 1 min at room temperature, and immediately resuspended in 2 ml RPMI1640 containing 100 U ml^−1^ penicillin and 10% FBS. Serial dilutions (100 µl of a 10-fold dilution) of the cell suspension were inoculated onto Eugon chocolate agar plates (Becton Dickinson), and the plates were incubated at 37°C for 3–4 days. CFU was calculated by multiplying the average number of colonies by 10 to account for the dilution factor.

### Bacterial Load In Lung, Spleen, and Liver in Mice Infected with SCHU P5 and SCHU P9

Three C57BL/6J mice in each group (7- to 12-week-old females; SLC, Inc. Shizuoka, Japan) were intranasally inoculated with 10 µl of 10^2^ and 10^6^ CFU bacteria under anesthesia with 0.08 µg medetomidine hydrochloride (Domitor; Orion Diagnostica, Espoo, Finland), 25 µg midazolam (Dormicum; AstellasPharma, Tokyo, Japan), and 0.3 µg butorphanol tartrate (Stadol; Bristol-Myers Squibb Company, Tokyo, Japan). Mice were sacrificed after anesthetization with isoflurane. Dissemination was quantified by harvesting lungs, spleens, and livers from the infected mice. The organs were homogenized in RPMI 1640, and the diluents were cultured onto Eugon chocolate agar plates (Becton Dickinson) at 37°C for 4 days. CFU was calculated by multiplying the average number of colonies by 10 to account for the dilution factor.

### Virulence of the *F. tularensis* SCHU Strains, pdpC Mutants, and Complemented Strains in Mice

To evaluate the virulence of SCHU P0, P5, and P9 strains in mice, four C57BL/6J mice in each group (seven- to twelve-week-old females; SLC, Inc. Shizuoka, Japan) were intraperitoneally or intranasally inoculated with 100 µl or 10 µl 10^1^–10^6^ CFU bacteria under anesthesia with 0.08 µg medetomidine hydrochloride (Domitor; Orion Diagnostica, Espoo, Finland), 25 µg midazolam (Dormicum; AstellasPharma, Tokyo, Japan), and 0.3 µg butorphanol tartrate (Stadol; Bristol-Myers Squibb Company, Tokyo, Japan). Clinical signs and body weight recovered after 14 days and 50% of lethal dose (LD_50_) were calculated. For investigation of virulence of *pdpC* knockout mutants and complemented strains in mice, C57BL/6J mice, four in each group, were inoculated intranasally (left nose) with 10^3^ and 10^6^ CFU of bacteria under anesthesia as described above. Clinical signs and body weight were recorded after 14 or 18 days. In case of extreme weight loss (>20%), mice were humanely sacrificed by isoflurane inhalation. All animal experiments were performed in a P3A facility.

## Results

### Reversion of Pathogenicity of the SCHU P0 Strain by Serial Passage in Mice

Mice infected intraperitoneally with 1×10^6^ CFU of SCHU P0 showed no severe clinical signs during the 3-week observation period (data not shown). We inferred that the low pathogenicity of SCHU P0 was caused by its extensive culturing on artificial media. Therefore, we conducted serial passages of SCHU P0 in mice, on the basis of previous reports stating that this regimen can restore the virulent phenotype [30]. After the 9th passage (SCHU P9), the bacteria became virulent. The scheme for SCHU P0, P5, and P9 isolation by *in vivo* passage is illustrated in figure 1A and described in detail in Materials and Methods. Mice intraperitoneally inoculated with 1×10^6^ CFU of SCHU P0 or P5 showed transient weight loss of ≤10% from 3 to 6 days post inoculation(dpi) and recovered completely by 10 dpi (Fig. 1B and D). In C57BL/6J mice intraperitoneally inoculated with 1×10^3^ CFU of SCHU P0 or P5, body weight loss and clinical signs were undetectable over a period of 2 weeks (Fig. 1C and E). All mice infected with SCHU P0 or P5 survived. In contrast, mice intraperitoneally inoculated with SCHU P9 showed rapid loss of up to 20% of their body weight and had to be sacrificed within 5 days (Fig. 1B–E). Mice intranasally inoculated with SCHU P9 10^2^ and 10^6^ CFU were also euthanized at 5 dpi as well as those with intraperitoneal infection (Fig. 1F and G). To evaluate the bacterial load in lungs, spleens, and livers, mice intranasally inoculated with SCHU P5 and P9 were sacrificed at the indicated incubation period, and the amounts of bacteria in the organs were determined (Fig. 1 H–J). The results showed that SCHU P9 could replicate and spread into spleens and livers. On the other hand, the growth of SCHU P5 in the lungs, spleens, and livers was limited. LD_50_ for SCHU P9 were calculated to be 5and 50 CFU after intraperitoneal and intranasal inoculation, respectively. These findings indicated that SCHU P0 and P5 were avirulent, but SCHU P9 was virulent in mice (Table 1).

**Table 1:** Bacterial mutants and complemented strains used in this study.

Strains/mutants	Description	Virulence
SCHU P0	*F. tularensis* maintained in the Ohara Research Laboratory	low
SCHU P5	Isolated after 5 passages in mice	low
SCHU P9	Isolated after 9 passages in mice	high
*pdpC knockout mutant*		
538 ins	Harboring mutation at nucleotide positions 538|539 using Targetron system derived from SCHU P9	low
1119 ins	Harboring mutation at nucleotide positions 1119|1120 using Targetron system derived from SCHU P9	low
2013 ins	Harboring mutation at nucleotide positions 2013|2014 using Targetron system derived from SCHU P9	low
*Complemented strain*		
P5+vec	SCHU P5 complemented with pNVU1 plasmid	low
P5+pA9	SCHU P5 complemented with pA9 plasmid encoding wild PdpC molecule	intermediate
P5+pA8	SCHU P5 complemented with pA8 plasmid encoding truncated PdpC molecule	low
538 ins+vec	538 ins complemented with pNVU1 plasmid	low
538 ins+pA9	538 ins complemented with pA9 plasmid encoding wild PdpC molecule	intermediate
538 ins+pA8	538 ins complemented with pA8 plasmid encoding truncated PdpC molecule	low

### Bacterial Growth on Artificial Media

SCHU P0, P5, and P9 strains were examined for their growth properties in chemically defined medium (CDM). All strains exhibited similar growth characteristics. Doubling times of SCHU P0, P5, and P9 during logarithmic growth phase (6–13 h after seeding) were 140, 135, and 134 min, respectively. Because bacterial growth is markedly influenced by iron and siderophore concentrations [31]–[33], growth rates were determined under limited iron concentrations (data not shown). No significant differences were observed under these conditions. Furthermore, these bacterial strains had similar growth in CDM of different pH (pH 2.8–7.8) (data not shown).

### Bacterial Growth in Cultured Phagocytes

**Figure 2:**
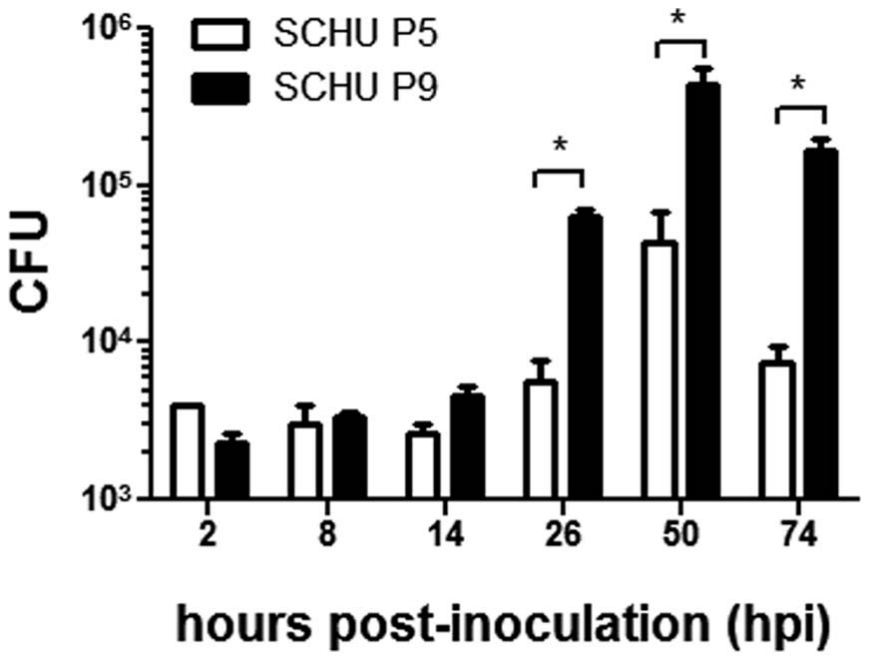
Bacterial growth in phagocytes. J774.1 cells were inoculated with SCHU P5 and P9 at an MOI of 10 and centrifuged and incubated for 1% Triton X-100 in CDM, and the intracellular CFU were measured in triplicate. Mean ± SEM of CFU are shown. The white and black columns indicate CFU of SCHU P5 and P9, respectively. Statistical significance was determined by Student’s *t* test (*p<0.05).

*F. tularensis* replicates in phagocytes such as macrophages and dendritic cells [34], [35]. Therefore, the ability of SCHU P5 and P9 strains to grow intracellularly in J774.1 cells was determined (Fig. 2). Cells inoculated with SCHU P5 and P9 at a multiplicity of infection (MOI) of 10 were cultured for 2, 8, 14, 26, 50, and 74 h, and the number of intracellular bacteria was measured. Equal levels of intracellular bacteria were observed in cells infected with SCHU P5 and P9 until 14 h post inoculation (hpi). In contrast, intramacrophage growth of SCHU P9 was significantly higher than that of SCHU P5 at 26 hpi and thereafter.

### SCHU Attenuation Caused by Frameshift Mutations in pdpC

To understand the molecular basis for difference in the virulence of differentially passaged bacterial strains, we determined the nucleotide sequences of the entire genomes of SCHU P0, P5, and P9. All sequence data obtained as short reads using an Illumina GA II were aligned to the reference sequence of *F. tularensis* subsp. *tularensis* SCHU S4 (Accession number: NC_0065870) [15]. These short reads were perfectly aligned to the SCHU S4 genomic sequence, except for a contiguous stretch corresponding to positions 1,759,066–1,759,167 (Table S2). Coverage of the SCHU S4 genome sequence was 99.995% in this analysis and the average depth per base in the SCHU P0, P5,and P9 sequences was 349, 297, and 355 reads, respectively (Table S2).

**Figure 3:**
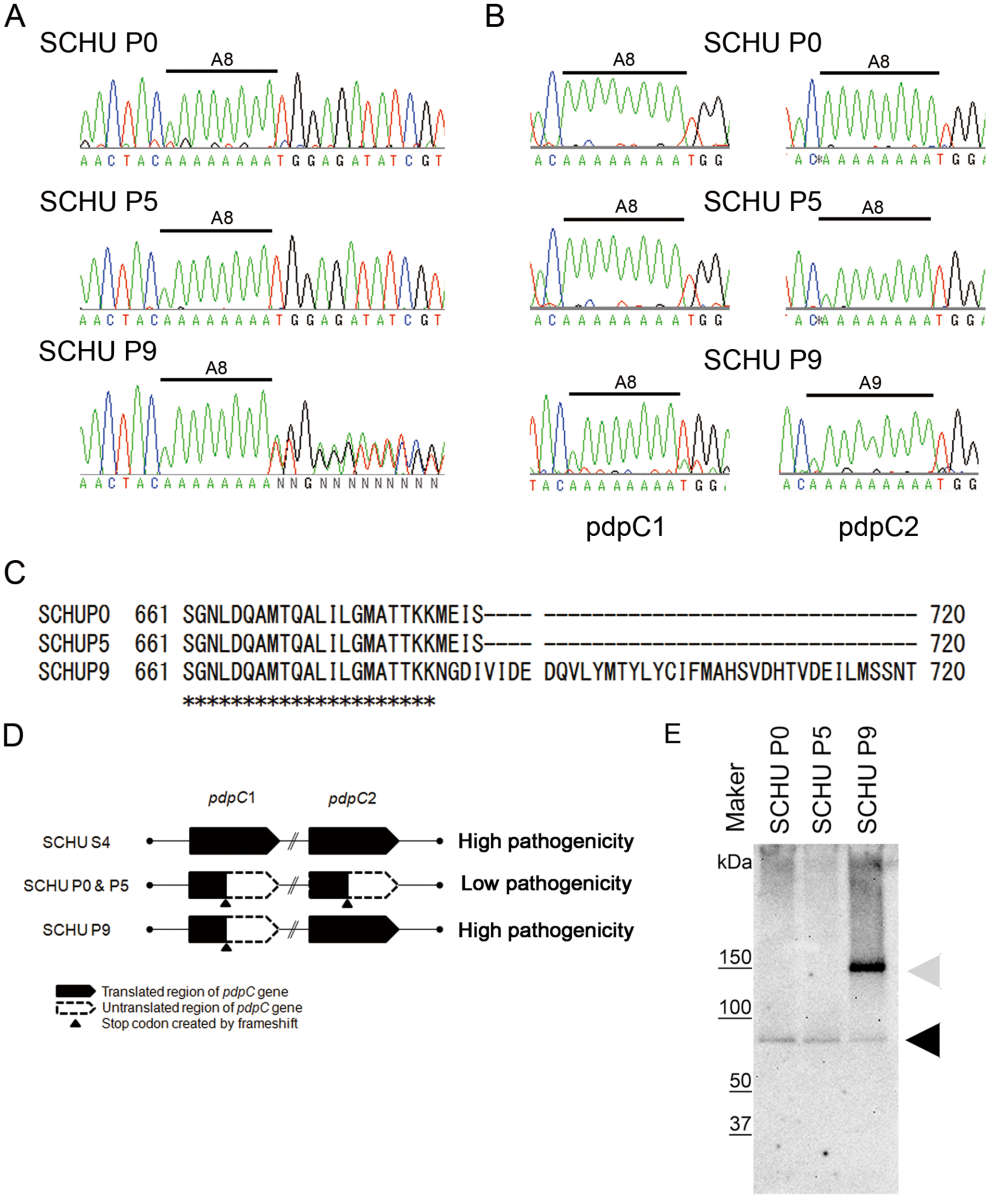
DNA sequencing analysis of the *pdpC* gene. (A) Mixtures of *pdpC1* and *pdpC2* DNAs amplified from the SCHU P0 (upper panel), P5 (middle panel), and P9 (lower panel) genomes were directly sequenced using Sanger’s method. The sequencing electropherograms of a region of *pdpC* from positions 2,031 to 2,056 are shown. (B) *pdpC1* (left panel) and *pdpC2* (right panel) were individually amplified by the long PCR method, and the amplicons were sequenced directly. The A tract found in the electropherograms is indicated as A8 or A9, as appropriate. (C) Deduced encoded amino acid sequences of PdpC2 from positions 661–720 obtained from SCHU P0, P5, and P9 were aligned. The asterisks and hyphens indicate the identical and untranslated amino acids, respectively. (D) Schematic representation of the structures of the *pdpC1* and *pdpC2* of *Francisella tularensis* SCHU S4, SCHU P0, SCHU P5, and SCHU P9. The translated and untranslated regions are indicated by black and white, respectively. The stop codons created by frameshift are indicated by arrowheads. (E) SCHU P0, P5, and P9 were grown in CDM and the lysates were subjected to western blot analyses using anti-PdpC polyclonal antibody [20]. Intact PdpC was observed only in SCHU P9. Bands corresponding to the expected sizes of the intact or truncated PdpCs are indicated by gray and black arrowheads, respectively.

We found 35 mutations in the SCHU P0 genome compared with the SCHU S4 genome. Because nucleotide substitutions found at 29 positions in the SCHU P0 sequence were shared by the SCHU P5 and P9 strains, these substitutions may not contribute to the increased virulence of SCHU P9 (Table S3). Among six other mutations, a nucleotide deletion in the SCHU P0 *pdpC* was also found in SCHU P5 (Table S4). In the *pdpC* of SCHU P0 and P5, an adenine (A) residue was missing from a 9-A residue stretch observed in SCHU S4. However, the *pdpC* of SCHU P9 appeared to comprise two different molecular species with an 8-A and a 9-A stretch, respectively. The read depths of these two species were 218 and 304, indicating that the molar ratio of these two species was nearly 1∶1 in SCHU P9 genome. This was confirmed by sequencing PCR amplicons of the *pdpC* using Sanger’s method (Fig. 3A). The data clearly show eight consecutive A residues followed by a thymidine (T) residue in the *pdpC* of SCHU P0 and P5 (Fig. 3A, upper and middle electropherograms), whereas a mixture of A and T was found at the same position in the SCHU P9 sequence (Fig. 3A, lower electropherogram).

Because *pdpC* is located in FPI, which is duplicated in the bacterial genome, two identical genes, *pdpC1* and *pdpC2*, are present. [14], [15]. Therefore, we tried to determine which one of SCHU P9 *pdpC* was mutated. To separately amplify the genes, we designed primer sets that were located outside FPI, so that *pdpC1* and *pdpC2* could be included in two independent amplicons. DNA fragments corresponding to the *pdpC1* or *pdpC2* regions were further amplified using PCR, and the resulting amplicons were sequenced directly. As depicted in Figure 3B, the relevant stretch of the *pdpC2* sequence of SCHU P9 contained nine A residues, whereas the *pdpC1* of SCHU P0, P5, and P9 as well as the *pdpC2* of SCHU P0 and P5 contained stretches of eight A residues. The lack of single A residue in a stretch of nine A residues in the *pdpC* resulted in a reading frameshift with a premature stop codon adjacent to the A tract, which led to production of a truncated protein. However, an A insertion in the *pdpC*2 of SCHU P9 restored the shifted frame to encode the wild-type protein observed in SCHU S4 (Fig. 3C and D). These results were consistent with the data obtained using the Illumina GA II as described above.

Western blot analyses were performed to confirm the sequence data described above. Antibodies directed against the N-terminal half [20] of PdpC recognized a band corresponding to a full-length PdpC (approximately 146 kDa) as well as a truncated PdpC molecule (approximately 75 kDa) in the lysates prepared from SCHU P9 cultures (Fig. 3E). In contrast, SCHU P0 and P5 expressed only the truncated PdpC (Fig. 3E).

### An Avirulent pdpC Mutant Derived from Virulent SCHU P9 in vitro

**Figure 4:**
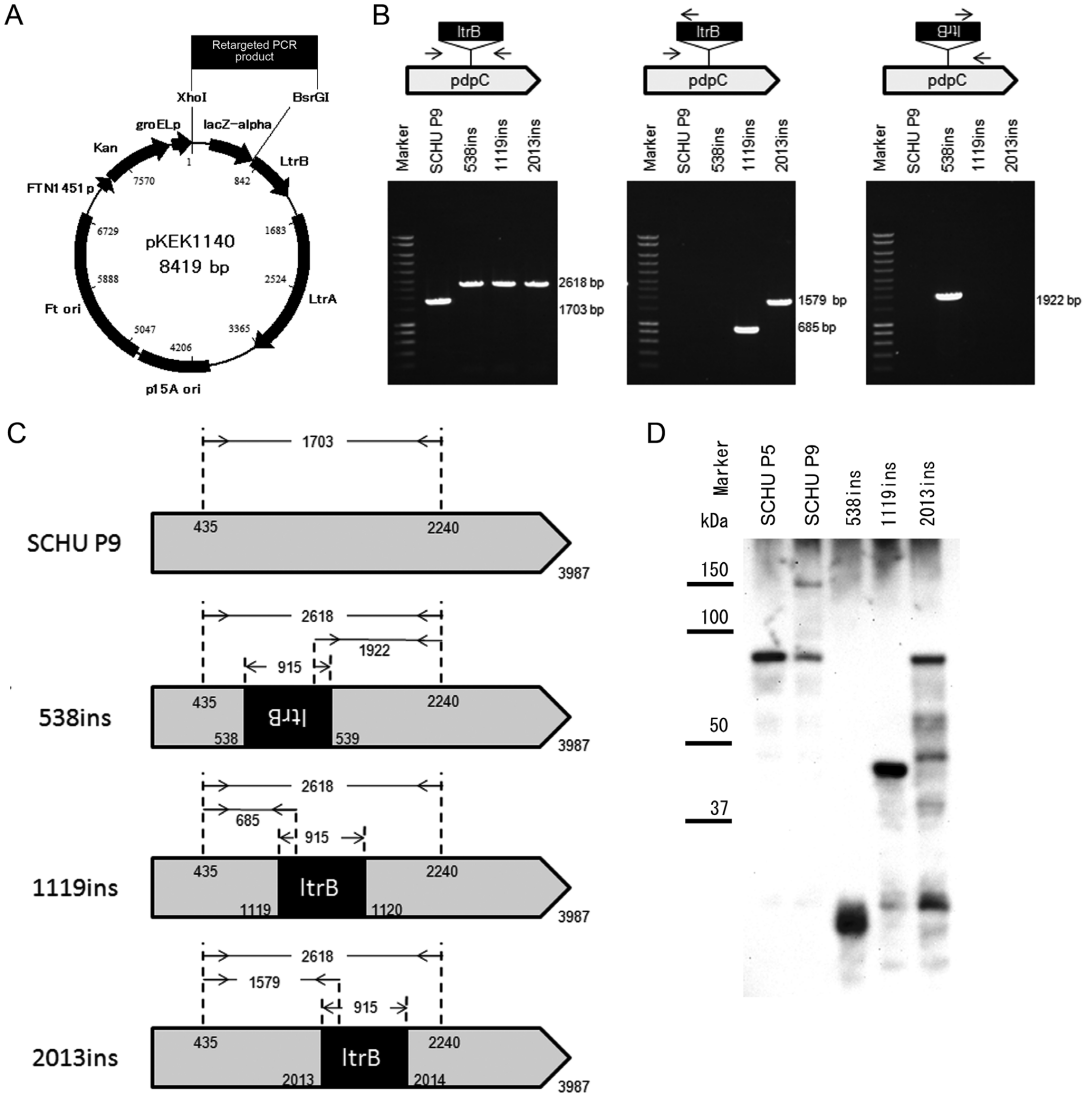
Construction of *pdpC* knockout mutants. The *pdpC* mutants were constructed using the TargeTron gene knockout system (Sigma-Aldrich) and pKEK1140 plasmid. (A) pKEK1140 plasmids were optimized for use in *Francisella tularensis* with the TargeTron gene knockout system. This plasmid consists of a plasmid origin for temperature-sensitive replication in *F. tularensis* (Ft ori), a plasmid origin in *E. coli* (p15A ori), an antibiotic resistance gene (Kan), FTN1451 promoter for Kan expressed in *F. tularensis* (FTN1451p), the RNP (*ltr*B_L1_ and LtrA) driven by the *F. tularensis groEL* promoter (groELp), and a *lacZ*′ within *ltr*B_L1_. To target specific *F. tularensis* genes for group II intron insertion, the *ltrB*_L1_ intron in pKEK1140 was retargeted by replacing lacZ′, which is located between the *Xho*I and *BsrG*I sites, with a 350-bp PCR product [27]. (B) The insertion in each *pdpC* mutant derived from SCHU P9 was confirmed by PCR. In the left panel, genomic DNA extracted from SCHU P9, 538 ins, 1119 ins, and 2013 ins mutants was amplified using sense (pdpC-435F) and antisense (pdpC-2204R) primers. In the middle and right panel, genomic DNA was amplified using insertion-and *pdpC*-specific primer pairs (pdpC-435F/EBS universal and EBS universal/pdpC-2204R). These amplicons and molecular weight marker were electrophoresed in a 0.7% agarose gel. (C) Summary of *pdpC* mutants generated in this study. *ltrB* introns were inserted into SCHU P9 *pdpC* positions 538|539, 1119|1120, and 2013|2014. The resultant mutants were designated 538 ins, 1119 ins, and 2013 ins mutants, respectively. Both duplicated *pdpC* were mutated by pKEK1140 plasmid in all mutants. (D) SCHU P5, SCHU P9, 538 ins, 1119 ins, and 2013 ins were grown in CDM and the lysates were subjected to western blot analysis was performed using anti-PdpC polyclonal antibody [20]. Intact PdpC was observed only in SCHU P9. Western blot analysis demonstrated that truncated forms of PdpC proteins with predicted molecular size (538 ins, approximately 20 kDa; 1119 ins, approximately 45 kDa; 2013 ins, approximately 75 kDa) were expressed in three *pdpC* mutants obtained.

To explore whether the recovery of a virulence of SCHU P9 was achievable by the insertion of an A residue in *pdpC2* leading to production of an intact PdpC molecule, we attempted to knockout *pdpC* in the SCHU P9 strain. Rodriguez *et al*. have recently developed a novel plasmid, pKEK1140, for rapid targeted gene disruption in *F. tularensis* utilizing mobile group II introns (TargeTron) [27]. We employed this method to construct *pdpC* knockout mutants derived from the virulent SCHU P9 strain. To identify the specific insertion sites on *pdpC*, the *pdpC* sequence of SCHU S4 was uploaded to the TargeTron Design Site (Sigma-Aldrich). Among many candidate insertion sites found, the nucleotide positions at 384|385, 538|539, 1119|1120, 2013|2014, 2806|2807 and 3245|3246 of in *pdpC*, which had low E-values, were selected. The intron PCR template was amplified using IBS, EBS1, EBS2, and EBS universal primers and ligated into pKEK1140 plasmid after treatment with the restriction enzymes *Xho*I and *Bsr*GI (Fig. 4A). SCHU P9 was transformed using these plasmids and cultured on chocolate Eugon agar plates (Becton Dickinson) containing kanamycin, and bacterial clones harboring mutations at nucleotide positions 538|539 (538 ins), 1119|1120(1119 ins), and 2013|2014 (2013 ins) were obtained. Genomic DNA extracted from these mutant strains was amplified using three primer pairs (Fig. 4B). The primer pair pdpC-435F/pdpC-2240R amplified a single 1,703-bp band when SCHU P9 DNA was used as a template, whereas 2,618-bp amplicons were obtained from the *pdpC* mutants using the same primer set (Fig. 4B, left panel), suggesting that both the duplicated *pdpC* were inactivated by *ltrB* insertion in these mutants.

When DNA templates prepared from the 1119 ins and 2013 ins mutants were subjected to PCR using a pdpC-435F/EBS universal primer pair, amplicons with the expected sizes were obtained (Fig. 4B, center panel); however, PCR using an EBS universal/pdpC-2204R primer pair yielded no amplicons (Fig. 4B, right panel). In contrast, an amplicon was obtained from 538 ins mutant DNA only using an EBS universal/pdpC-2204R primer pair (Fig. 4B, right panel). These results confirmed that *ltrB* was inserted into specific sites in the respective mutants (Fig. 4C). By semiquantitative RT-PCR using primers directed toward 5′ proximal region of *pdpC*(56–456), we found that an equal level of *pdpC* was transcribed regardless of the presence of the intervening introns (Fig. S1). However, western blot analysis demonstrated that truncated forms of PdpC proteins with predicted molecular size (538 ins, approximately 20 kDa; 1119 ins, approximately 45 kDa; 2013 ins, approximately 75 kDa) were expressed in three *pdpC* mutants obtained using TargeTron technology (Fig. 4D).

### Pathogenicity of pdpC Mutants

**Figure 5:**
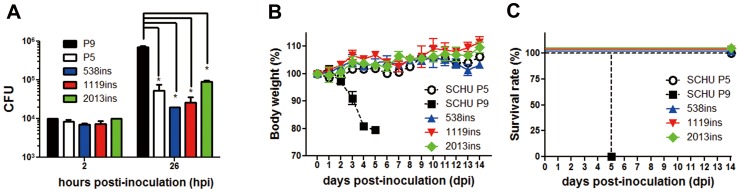
Complementation of avirulent phenotype of SCHU P5 and *pdpC* mutant 538 ins with wild type *pdpC*. The pathogenicity of the 538 ins, 1119 ins, and 2013 ins mutants was analyzed *in vitro* and *in vivo*. (A) J774.1 cells were inoculated with SCHU P5, SCHU P9, and the three mutants, at an MOI of 10, as described in Figure 2. After 2 and 26 h incubation, cells were washed and solubilized in 0.1% Triton X-100 in CDM. Intracellular CFU of triplicate samples were measured. Mean ± SEM of CFU are shown. Statistical significance was determined by ANOVA (*p<0.001). (B and C) Four C57BL/6J mice from each group were infected intranasally with 1×10^6^ CFU of the SCHU P5 (black circle), SCHU P9 (black square), 538 ins (blue triangle), 1119 ins (red triangle), and 2013 ins (green diamond) mutants. Their body weights and clinical signs were measured and observed for 14 days. The survival rates (B) and percentage body weights (C) are shown.

The ability to grow in phagocytic cells was compared between the *pdpC* mutants and the parental strain SCHU P9. SCHU P5 was also included in the study. The number of intracellular bacteria in J774.1 cells measured at 26 hpi was significantly greater in SCHU P9 infected cells as compared with that of all other bacterial strains including mutants (Fig. 5A). These results suggested that *pdpC* encoding the intact PdpC was crucial for the intracellular growth.

We next examined these strains for the difference in pathogenicity in mice. Intranasally inoculated mice with 1×10^6^ CFU of SCHU P9 showed a rapid 20% reduction in body weight. Because infected mice suffered from severe diseases, they had to be euthanized within 5 days as shown in Figure 1 (Fig. 5B and C). However, mice infected with either SCHU P5 or three mutants survived without any apparent clinical signs and weight loss (Fig. 5B and C). These results indicated that only disruption of *pdpC* was sufficient to attenuate SCHU P9 (Table 1).

### Complementation of Avirulent Phenotype of the Bacteria with Wild Type pdpC

**Figure 6:**
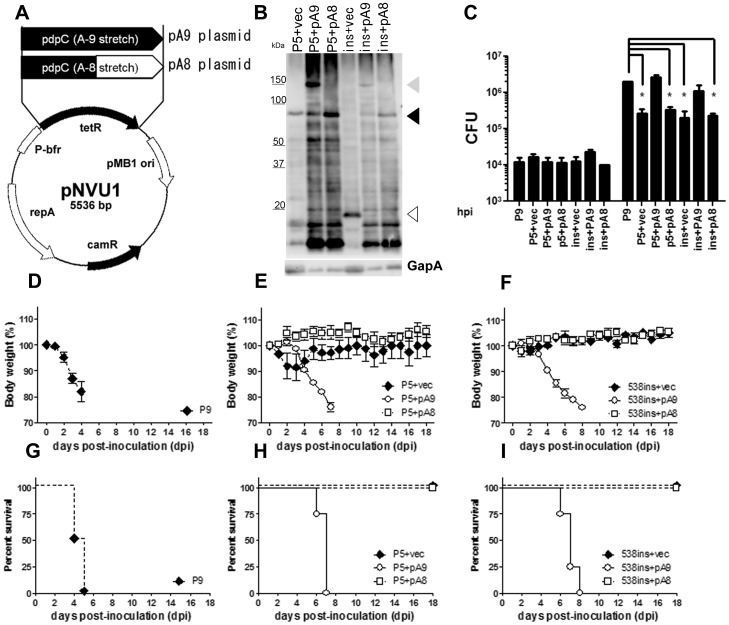
Complementation analysis in an virulent strain and in the *pdpC* mutant. (A) The genetic maps of pNVU1, pA9, and pA8 plasmids are shown. The pNVU1 plasmid constituted the tetracycline resistance gene (tetR) under control of *Francisella bfr* promoter (P-bfr) [28] and a partial sequence of pOM5 [29] containing pMB1 ori (pMB1), which is essential replication in *E. coli*, a chloramphenicol resistance gene (camR), and the RepA gene, which is essential for replication in *Francisella*. When tetR in pNVU1 plasmid was replaced with *pdpC* containing a 9-A stretch (pA9), this plasmid generated full length PdpC, while the pA8 plasmid expressed truncated PdpC due to the frameshift. (B) Expression of PdpC in the SCHU P5 and 538 ins mutant complemented with pA9, pA8 and pNVU1 (P5+pA9, P5+pA8, P5+vec and ins+pA9, ins+pA8, ins+vec, respectively) were analyzed using western blot. Each complemented bacteria was grown in CDM and the bacterial lysates were subjected to western blot analysis using anti-PdpC polyclonal antibodies [20] and anti-GapA polyclonal antibodies as a loading control. Wild type PdpC(gray arrowhead) was only observed in pA9-complemented bacteria (P5+pA9 and ins+pA9), whereas only truncated PdpC (black arrowhead) was detected in pA8-complemented strains (P5+pA8 andins+pA8). Ins+vec expressed further truncated protein (white arrowhead). (C) J774.1 cells were inoculated with bacteria at an MOI of 10. The intracellular bacteria were quantified at 2 and 26 h post-inoculation (hpi). Bars represent mean ± SEM of triplicate samples. Statistical significance was determined by ANOVA (*p<0.001). (D–I) Four C57BL/6J mice from each group were infected intranasally with 1×10^6^ CFU of SCHU P9 (D and G), P5 (E and H), 538 ins (F and I), and pA9- or pA8-complemented bacteria (E, F, H, and I). Their body weights and clinical signs were measured and observed for 18 days. The percentage body weights (D, E, and F) and survival rates (G, H, and I) are shown in the upper and lower panels, respectively.

Because a truncation or knockout of *pdpC* resulted in attenuation of the bacteria, we tested if the avirulent phenotype was reversed or complemented with a plasmid expressing wild type *pdpC*. We constructed pA9 and pA8 plasmids carrying wild type and truncated *pdpC*, respectively, under control of the *Francisella bfr* promoter [28] (Fig. 6A). Avirulent SCHU P5 and 538 ins mutants were transformed with pA9 or pA8 plasmids. Western blot (Fig. 6B) analysis showed that a 146 kDa protein corresponding to wild type PdpC was detected when SCHU P5 or 538 ins was transformed with pA9 plasmid (gray arrowhead, Fig. 6B). In contrast, the bacteria complemented with pA8 expressed a 75 kDa protein corresponding to the truncated form of PdpC (lane P5+pA8 and ins+pA8; black arrowhead, Fig. 6B).

We next evaluated the intracellular growth of the *pdpC*-complemented bacteria in J774.1 cells together with wild strains (Fig. 6C). Robust bacterial growth was observed upon infection of J774.1 cells with strains complemented with pA9 plasmid. In contrast, as expected, pA8 plasmid did not confer such an effect.

C57BL/6J mice were intranasally inoculated with 1×10^6^ CFU of each bacteria to see whether the reversion of pathogenicity was achieved by transformation of avirulent strains with pA9 plasmid (Fig. 6E–I). Mice inoculated with SCHU P5 and 538 ins mutant transformed with blank pNVU1 plasmid (referred to as P5+vec and 538 ins+vec, respectively) showed neither clinical signs nor loss of body weight (Fig. 6E, F, H, and I, black diamond), coinciding with observation made for parental SCHU P5 and 538 ins mutant (Fig. 1 and Fig. 5). pA8-complemented bacteria also failed to induce clinical signs and weight loss in mice, and all mice survived (Fig. 6 E, F, H and I, open square). In contrast, mice inoculated with pA9-complemented bacteria (Fig. 6E, F, H, and I, open circle) showed severe clinical signs and failed to survive, although it took two or three more days until these mice succumbed to the infection as compared with SCHU P9 infection. These data indicated that intracellular growth and virulence in mice of the bacteria were partially complemented to wild-type PdpC expressed through the pA9 plasmid (Table 1).

## Discussion

Details of molecular mechanisms involved in the pathogenicity of *F. tularensis*, which not only poses a threat to public health under normal conditions but can also be weaponized, have been the focus of quite a few studies. A gene knockout strategy to introduce random mutations is useful for analyzing gene function or screening for genes essential for the manifestation of high pathogenicity. *Francisella* mutants have been created by this technique, and their biological properties have been assessed by infecting phagocytes *in vitro* [22], [36]–[39]. In this study, we obtained a virulent strain from an attenuated SCHU strain by serial passages in mice, and attempted to identify the pathogenicity-determining genes by comparing whole genome sequences of the attenuated and virulence reverted bacteria.

SCHU P0 stored at the Ohara Research Laboratory was obtained as the SCHU strain from the United States. The pathogenicity of this strain was estimated using mice by Sato *et. al.* [40]. According to this report, the SCHU P0 was already attenuated. Comparing the genomic sequences between SCHU P0 and Schu S4, 29 single nucleotide polymorphisms (SNPs: 4 synonymous, 4 nonsense, 19 nonsynonymous, 2 noncoding) were found (Table S3 and S4). However, FRAN030 [41], which is genetically closely related to SCHU S4, possessed 89 SNPs. These evidences strongly supported that SCHU P0 derived from the original SCHU isolate or from the Schu S4 subclone, even though, it remains unclear precisely which genetic changes are responsible for the attenuation of SCHU P0.

We found that attenuated *F. tularensis* SCHU P0 became virulent in mice after the 9th passage, but not after the 5th passage. Taking advantage of recent advances in high-throughput nucleotide sequencing, we determined complete genomic sequences of the SCHU P0, P5, and P9 strains and compared them with that of the virulent SCHU S4 strain. The analysis identified a single nucleotide deletion in the *pdpC1* and *pdpC2* of SCHU P0 and P5, which resulted in the expression of a truncated PdpC protein. SCHU P9 retained the deletion in the *pdpC1*; however, the deletion was reverted by a insertion of A residue in the *pdpC2*. Therefore, SCHU P9 encoded both truncated and wild-type PdpC proteins, which were actually demonstrated by western blot analysis (Fig. 3E). Of interest, attenuated SCHU P0 carried the identical single nucleotide deletion in both copies of *pdpC* that were likely acquired by continuous passage in artificial media. Although it could not be experimentally demonstrated, it would be possible that a single deletion event in one *pdpC* occurred during serial passages in artificial media and was transmitted to the other allele by homologous recombination during further passages in the artificial media.

By comparing SCHU P9 and Schu S4, we also noticed the pathogenic effects of another 29 SNPs and 5 insertion/deletion sites (Table S3 and S4). We examined whether the pathogenicity in SCHU P9 was different from that in Schu S4. However, it was difficult to obtain Schu S4 strain from other laboratories because the transfer of virulent bacteria is strictly restricted in Japan, we tried to compare the pathogenicity of SCHU P9 and Schu S4 indirectly. Mice inoculated with 10^2^ CFU SCHU P9 were sacrificed at 1–4 dpi and then the CFU in lungs, livers, and spleens were enumerated (Fig. 1H–J). We detected 10^7^–10^8^ CFU in the livers and spleens. These data strongly suggested that SCHU P9 was a virulent strain because the bacterial loads were at the same levels of those in Schu S4 infection [42], [43].

Sjodin *et al.* [9] compared the genomic sequence of attenuated *F. tularensis* FSC043 that was independently obtained by serial passages *in vitro* with that of *F. tularensis* SCHU S4. They found four deletions resulting in the disruption of genes coding for metal–ion transporter proteins (locus_tag; FTT_0615c), *pdpC1* (FTT_1354), *pdpC2* (FTT_1709), and RD18 (intergenic region from FTT_0918 to FTT_0919) of the attenuated *F. tularensis* FSC043. Because only *pdpC* was mutated in both attenuated strains, SCHU P0 and FSC043, it is possible that *pdpC* alone determined the virulence of each strain, although we cannot rule out the possibility that the metal–ion transporter protein and RD18 are associated with bacterial virulence.

It is unclear which genetic changes are responsible for the attenuation of SCHU P0. However, FSC043, an attenuated derivative of Schu S4, also has deletion in *pdpC1* and *pdpC2* [44]. In addition, SCHU P0 and P9 has only one nucleotide difference in *pdpC2*, resulting in reversion of truncation of the PdpC protein in SCHU P9, and SCHU P9 showed comparable level of pathogenicity to Schu S4 as shown in this study (Fig. 1H–J). Furthermore, the *pdpC* mutants of Schu S4 were defective in replication in J774.1 cells and showed avirulent phenotype in mice [24]. These suggested that *pdpC* is responsible for attenuation of SCHU P0. SCHU P9 is a clone of the 9th passage, so we have determined the sequence of *pdpC2* region by direct sequencing of the 9th passage homogenates and found the reversion of *pdpC2* was dominant.

Evidence suggests that phagocytes are the major replication sites for this bacterium *in vivo* [45], [46]. On invading its host, *F. tularensis* readily escaped from the phagosome and replicated in the cytoplasm of infected cells. We found a significant difference between SCHU P5 and P9 strains in intracellular bacterial growth in J774.1 cells (Fig. 2). Our results here unambiguously showed that the *pdpC* is an essential element determining *F. tularensis* pathogenicity (Fig. 2, 3 and 4).

In this study, mRNA expression levels in *pdpC* mutants were analyzed and no apparent differences in mRNA expression detectable by primers designed to amplify the 5′ region of *pdpC* was observed (Fig. S1). Long *et al.* recently derived a *pdpC*-608 mutant from *F. tularensis* SCHU S4 harboring a mutation at nucleotide positions 608|609 [24]. They showed that transcription of *pdpC* was ablated in the mutant [24]. However, their results were found to be inconsistent with our findings. Although the reason for the difference in mRNA expression levels between pdpC-608 and our three *pdpC* mutants is unclear, it may be due to different insertion sites between the bacterial strains. In mutants, expression of truncated PdpC protein with predicted molecular size was observed by western blotting (Fig. 4D).

Low pathogenicity of SCHU P5 and *pdpC* mutant was restored by complementation with pA9 plasmid expressing the wild type PdpC molecule, but not with pA8 plasmid expressing the truncated PdpC molecule (Fig. 6). It was recently reported that *pdpC* mutants derived from *F. tularensis* Schu S4 was defective in replication in monocyte-derived macrophage and J774.1 cells and showed avirulent phenotype in mice as well [24], [25]. Lindgren *et al.* also reported similar findings using pdpC disrupted mutants derived from *F. tularensis* subsp. *holarctica* LVS. Our study strongly supported their results. On the contrary several previous reports have described that *F. novicida* did not require *pdpC* for intracellular proliferation in insect cell lines and fruit flies [22], [23]. The conflicting results may be due to the differences in the relationships between the bacterial strains and their hosts.

Analysis of the *pdpC* sequence data using BLAST and PROSITE [44] failed to find any functional similarities to sequences of other proteins present in the database. When the isoelectric points (p*I*) and molecular weights (M_r_) were calculated *in silico* using DNASIS pro (Hitachi software, Tokyo, Japan), PdpC was predicted to be highly basic (p*I* = 9.440) and relatively large (M_r_ = 156,186). When we examined the database for *F. tularensis* SCHU proteins with similar characteristics, i. e., p*I* = 9.0–9.6 and M_W_ = 1200000–180000, ATP-dependent helicase (HrpA) emerged. DNA-directed RNA polymerase subunit beta, eukaryotic translation initiation factor 3 subunit A, translation initiation factor IF-2, regulator of G-protein signaling protein-like telomerase reverse transcriptase, and other proteins also exhibited significant similarity. Most of these proteins possess typical nucleic acid-interacting protein motifs, suggesting that PdpC may be included in this category, although further investigation is necessary. Beside the evidence provided by sequence analysis, information regarding the function of PdpC is limited. Cheung *et al*. [47] showed that the erythromycin allelic replacement mutant of *pdpC* was slightly attenuated in terms of growth within the J774.1 cell line as well as in chicken embryos, and that complementation partially restored virulence. Moreover, it was suggested that PdpC may play a role in cytoskeletal rearrangement by altering host cell signaling [47].

In this study, virulence reverted SCHU P9 was shown to express both wild type and truncated form of PdpC proteins. Furthermore, SCHU P5 and the *pdpC* mutant that recovered virulence by transformation with pA9 plasmid expressed both wild type and truncated form of PdpC proteins, indicating that the truncated PdpC protein did not exert dominant negative effect to the wild type protein.

In this study, the experimental protocol was to obtain “virulent reverted” bacterium from the attenuated *Francisella tularensis* strain, SCHU P0. The NIID biosafety committee approved the experiment since the protocol did not correspond to a dual use research of concerning National Science Advisory Board for Biosecurity (NSABB) in terms of enhancement of virulence and susceptibility of a host population of the bacterium. In this experiment, virulent SCHU P9 was obtained from the attenuated SCHU P0 by *in vivo* passages in mice, which were highly susceptible for *Francisella tularensis*. However, the virulence of SCHU P9 was equal to or less than wild type of *Francisella tularensis* subspecies *tularensis* strains.

Since the virulence reverted SCHU P9 was easily isolated from the attenuated strain, it was suggested that the attenuated live vaccine strains obtained by extensive *in vitro* passage might have a risk of virulence reversion during replication *in vivo*. On the other hand, attenuated phenotype of the *pdpC* knockout mutants produced by the intron insertion is likely to be stable. Therefore, the *pdpC* knockout mutants are good candidates of live vaccine strains.

In conclusion, comparison of virulent and attenuated *F. tularensis* strains identified a single nucleotide in *pdp*C*2* which is responsible for attenuation of virulence. Analysis of *pdpC* knock out mutants along with trans-complementation experiments explicitly demonstrated that *pdpC* is predominant gene contributing to the pathogenicity of *F. tularensis.*

## Supporting Information

Figure S1 **mRNA expression of pdpC and pdpE genes were evaluated by semiquantitative RT-PCR.** Primers were designed to amplify pdpC (pdpC/56–456), pdpE (pdpE/254–540), and gapA(gapA/322–459). The bars under a gene map indicate the amplified gene region. Primers for gapA were added to all reactions as an endogenous control. Each RNA sample is divided as an RT+ sample and an RT− sample (negative control).
(TIF)

Table S1 **Primers used in this study.**
(DOCX)

Table S2 **The summary of whole genome sequencing analysis by Illumina Genome Analyzer II.**
(DOCX)

Table S3 **Single nucleotide mutation position and mix population rate among P0, P5 and P9 based on SCHU S4 genome sequence.**
(DOCX)

Table S4 **Insertion and deletion position and mix population rate among P0, P5 and P9 based on SCHU S4 genome sequence.**
(DOCX)
