# Supplementary figures and images for: Role of Pathogenicity Determinant Protein C (PdpC) in Determining the Virulence of the Francisella tularensis Subspecies tularensis SCHU
Source: PLoS One. 2014 Feb 18;9(2):e89075. doi: 10.1371/journal.pone.0089075 (PMC3928404; doi:10.1371/journal.pone.0089075)

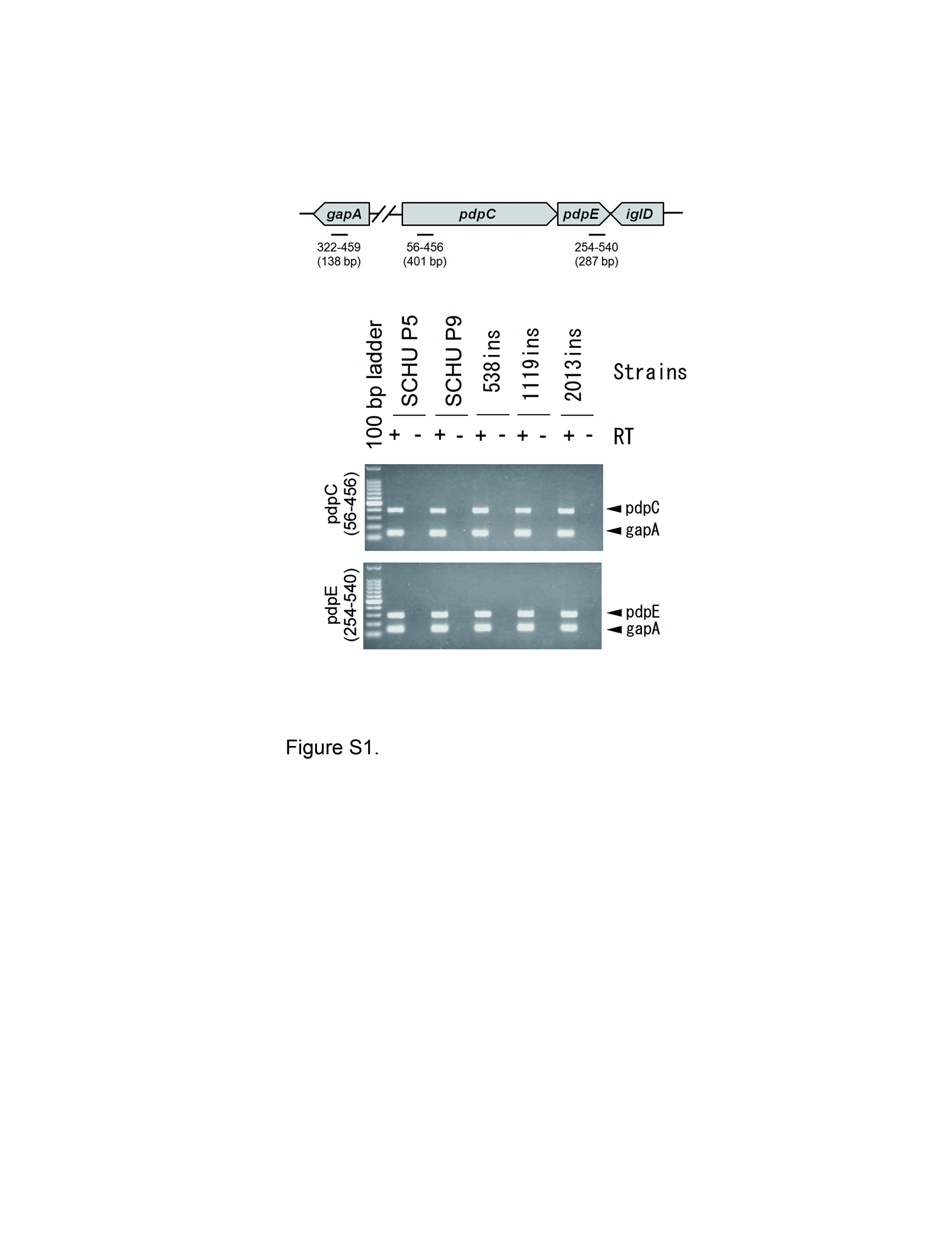

Supplement: Figure S1 — mRNA expression of pdpC and pdpE genes were evaluated by semiquantitative RT-PCR. Primers were designed to amplify pdpC (pdpC/56–456), pdpE (pdpE/254–540), and gapA(gapA/322–459). The bars under a gene map indicate the amplified gene region. Primers for gapA were added to all reactions as an endogenous control. Each RNA sample is divided as an RT+ sample and an RT− sample (negative control). (TIF) [file pone.0089075.s001.tif]
