# Supplementary material for: Role of Pathogenicity Determinant Protein C (PdpC) in Determining the Virulence of the Francisella tularensis Subspecies tularensis SCHU
Source: PLoS One. 2014 Feb 18;9(2):e89075. doi: 10.1371/journal.pone.0089075 (PMC3928404; doi:10.1371/journal.pone.0089075)
Supplement: Table S1 — Primers used in this study. (DOCX) [file pone.0089075.s002.docx]

Table S1. Primers used in this study.

| **Primer name** | **5'->3' primer sequence** | **Nuclotide position**  **(NC_006570)** |
| --- | --- | --- |
| **Primers to confirm pdpC nucleotide sequences** | | |
| PI1F | TCATAATTCGTAACTCACAGAAACA | 1,373,404 - 1,373,428 |
| PI1R | CAACGCTATGAGAGTGGTGAGT | 1,410,226 - 1,410,247 |
| PI2F | CAAAGAAGCTGCTCCTGCTA | 1,765,833 - 1,765,852 |
| PI2R | GCTAGGAAGTGGGTTGGATG | 1,802,074 - 1,802,093 |
| FT-1394871-1394879-F | TCATCTCAAATCAAGCCAAGC | 1,394,491 -　1,394,511 1,787,826 - 1,787,846 |
| FT-1394871-1394879-R | TCGACACTATGTGCCATGAAA | 1,394,937 - 1,394,957 1,788,272 - 1,788,292 |
| **Primers to construct pdpC mutants** | | |
| EBS Universal | CGAAATTAGAAACTTGCGTTCAGTAAAC |  |
| pdpC-538IBS^a^ | AAAACTCGAGATAATTATCCTTATTTTTCCCACTAGTGCGCCCAGATAGGGTG |  |
| pdpC-538EBS1d^b^ | CAGATTGTACAAATGTGGTGATAACAGATAAGTCCCACTAACTAACTTACCTTTCTTTGT |  |
| pdpC-538EBS2 | TGAACGCAAGTTTCTAATTTCGGTTAAAAATCGATAGAGGAAAGTGTCT |  |
| pdpC-1119IBS^a^ | AAAACTCGAGATAATTATCCTTAAATATCAACCCGGTGCGCCCAGATAGGGTG |  |
| pdpC-1119EBS1d^b^ | CAGATTGTACAAATGTGGTGATAACAGATAAGTCAACCCGAATAACTTACCTTTCTTTGT |  |
| pdpC-1119EBS2 | TGAACGCAAGTTTCTAATTTCGATTATATTTCGATAGAGGAAAGTGTCT |  |
| pdpC-2013IBS^a^ | AAAACTCGAGATAATTATCCTTAATGACCCAGGCAGTGCGCCCAGATAGGGTG |  |
| pdpC-2013EBS1d^b^ | CAGATTGTACAAATGTGGTGATAACAGATAAGTCCAGGCATTTAACTTACCTTTCTTTGT |  |
| pdpC-2013EBS2 | TGAACGCAAGTTTCTAATTTCGGTTGTCATCCGATAGAGGAAAGTGTCT |  |
| pdpC-435F | TCAAAACATCATCCATTTAGCA | 1,393,269-1,393,290  1,786,604-1,786,625 |
| pdpC-2240R | GAAAGTTTAAATACAGGTCTAGCAAAA | 1,395,048-1,395,074  1,788,383-1,788,409 |
| **Primers to construct complement plasmid^C^** | | |
| pNVU1-s | ggtcgacccgggTGTCAGACCAAGTTTACTCATATATAC | template: pOM5: ref. Pomerantsev *et al.* |
| pNVU1-a | gatcctcgagTAAATCCATGTACTTAAATAAGTACTTAAAG | template: pOM5: ref. Pomerantsev *et al.* |
| Tet-s | tcccgggcctaggataaattaaggaggtacatATGAAATCTAACAATGCGCTCATC | template: pBR322 |
| Tet-a | acacccgggtcgacCTAGGTCGAGGTGGCCCG | template: pBR322 |
| Pbfr-s | atttactcgagGATCCATACCCATGATGGTTATTATTGCC |  |
| Pbfr-a | atcctaggcccggGATCAATAATTTCTTGTTTATTTTCTAATTG |  |
| pdpC-s | tcccgggcctagGATAAATTAAGGAGGTACATATG |  |
| pdpC-a | acacccgggtcgacCTATGATGATATTTTTTTAAAAAAGTCTGATTTATATAAG |  |

a HindIII restriction site on IBS primer that designed by the Sigma-Aldrich computer-based TargeTron algorithm was replaced to XhoI restriction site　(underline).

b The underline indicate the BsrGI restriction site.

c The undreline indicate the 15 bp overlap region for In-Fusion Cloning. The large letters indicate annealing site to templates.
