# Supplementary material for: Role of Pathogenicity Determinant Protein C (PdpC) in Determining the Virulence of the Francisella tularensis Subspecies tularensis SCHU
Source: PLoS One. 2014 Feb 18;9(2):e89075. doi: 10.1371/journal.pone.0089075 (PMC3928404; doi:10.1371/journal.pone.0089075)
Supplement: Table S2 — The summary of whole genome sequencing analysis by Illumina Genome Analyzer II. (DOCX) [file pone.0089075.s003.docx]

**Table S2. The summary of whole genome sequencing analysis by Illumina Genome Analyzer II.**

| Strain name | Numbers of total read  (single 80mer) | Mapping read (%) | Average depth (/base) | No mapping region on SCHU S4 genome | Coverage of SCHU S4 genome (%) |
| --- | --- | --- | --- | --- | --- |
| SCHU P0 | 8,487,918 | 97.3 | 348.924 | 1759066..1759167 | 99.995 |
| SCHU P5 | 7,929,949 | 88.7 | 297.191 | 1759068..1759167 | 99.995 |
| SCHU P9 | 8,677,478 | 96.9 | 355.379 | 1759066..1759167 | 99.995 |
