# Supplementary material for: Role of Pathogenicity Determinant Protein C (PdpC) in Determining the Virulence of the Francisella tularensis Subspecies tularensis SCHU
Source: PLoS One. 2014 Feb 18;9(2):e89075. doi: 10.1371/journal.pone.0089075 (PMC3928404; doi:10.1371/journal.pone.0089075)
Supplement: Table S3 — Single nucleotide mutation position and mix population rate among P0, P5 and P9 based on SCHU S4 genome sequence. (DOCX) [file pone.0089075.s004.docx]

**Table S3. Single nucleotide mutation position and mix population rate among P0, P5 and P9 based on SCHU S4 genome sequence.**

| Genomic postion*^a^* | Strand of described nucleotide | Detected mutation*^b^* | | Type of nucleotide substitution | Type of amino acid substitution | Amino acid substitution |  | Read depth*^c^* | | | | | | | | | | |  | Genetic information on variation | | |
| --- | --- | --- | --- | --- | --- | --- | --- | --- | --- | --- | --- | --- | --- | --- | --- | --- | --- | --- | --- | --- | --- | --- |
|  |  | SCHU S4 | P0, P5 and P9 |  |  |  |  | P0 | | |  | P5 | | |  | P9 | | |  | locus tag | gene name | product |
|  |  |  |  |  |  |  |  | Identical nucleotide with SCHU S4 | Variant nucleotide | Variant Frequency |  | Identical nucleotide with SCHU S4 | Variant nucleotide | Variant Frequency |  | Identical nucleotide with SCHU S4 | Variant nucleotide | Variant Frequency |  |  |  |  |
| 80034 | + | GA**A** | GA**C** | Transversion | nonsynonymous | Glu 3 Asp |  | 377 | 86 | 19% |  | 263 | 74 | 22% |  | 279 | 52 | 16% |  | FTT_0077 | sucB | dihydrolipoamide succinyltransferase component of 2-oxoglutarate dehydrogenase complex |
| 107425 | - | AG**C** | AG**A** | Transversion | nonsynonymous | Ser 43 Arg |  | 165 | 24 | 13% |  | 182 | 55 | 23% |  | 224 | 57 | 20% |  | FTT_0103c | - | hypothetical protein |
| 132117 | + | **A**GG | **T**GG | Transversion | nonsynonymous | Arg 378 Trp |  | 236 | 49 | 17% |  | 191 | 57 | 23% |  | 206 | 40 | 16% |  | FTT_0121 | uvrD | DNA helicase II |
| 136155 | + | **A** | **C** | Transversion | - | - |  | 185 | 29 | 14% |  | 165 | 19 | 10% |  | 54 | 20 | 27% |  | Intergenic region (FTT_0121 - 0125) |  |  |
| 143455 | + | T**G**G | T**A**G | Transition | nonsense mutation | Trp 497 Stop |  | 0 | 279 | 100% |  | 0 | 257 | 100% |  | 0 | 308 | 100% |  | FTT_0130 | glpK | glycerol kinase |
| 161920 | + | **A**TT | **C**TT | Transversion | nonsynonymous | Ile 954 Leu |  | 279 | 83 | 23% |  | 226 | 61 | 21% |  | 226 | 44 | 16% |  | FTT_0145 | rpoC | DNA-directed RNA polymerase, beta subunit |
| 163196 | + | T**C**G | T**A**G | Transversion | nonsense mutation | Ser 1379 Stop |  | 0 | 215 | 100% |  | 0 | 243 | 100% |  | 0 | 244 | 100% |  | FTT_0145 | rpoC | DNA-directed RNA polymerase, beta subunit |
| 195933 | + | TA**C** | TA**A** | Transversion | nonsense mutation | Tyr 9 Stop |  | 114 | 34 | 23% |  | 196 | 34 | 15% |  | 267 | 37 | 12% |  | FTT_0180 | - | putative acyltransferase |
| 241178 | + | **G** | **T** | Transversion | - | - |  | 242 | 43 | 15% |  | 181 | 27 | 13% |  | 168 | 49 | 23% |  | Intergenic region (FTT_0223c - 0226c) |  |  |
| 267203 | + | A**T**A | A**A**A | Transversion | nonsynonymous | Ile 36 Lys |  | 157 | 17 | 10% |  | 149 | 9 | 6% |  | 64 | 22 | 26% |  | FTT_0251 | ilvE | branched-chain amino acid aminotransferase protein (class IV) |
| 281395 | + | G**A**T | G**C**T | Transversion | nonsynonymous | Asp 276 Ala |  | 193 | 24 | 11% |  | 156 | 26 | 14% |  | 82 | 25 | 23% |  | FTT_0266 | - | ABC transporter, ATP-binding protein |
| 292794 | - | **G**TG | **A**TG | Transition | nonsynonymous | Val 244 Met |  | 0 | 336 | 100% |  | 0 | 274 | 100% |  | 0 | 325 | 100% |  | FTT_0279c | cydA | cytochrome d terminal oxidase, polypeptide subunit I |
| 300160 | + | AT**A** | AT**C** | Transversion | synonymous | - |  | 183 | 11 | 6% |  | 224 | 13 | 5% |  | 117 | 29 | 20% |  | FTT_0285 | cyoE | Protoheme IX farnesyltransferase |
| 464457 | - | **G**GC | **T**GC | Transversion | nonsynonymous | Gly 297 Cys |  | 81 | 11 | 12% |  | 77 | 8 | 9% |  | 37 | 11 | 23% |  | FTT_0448c | glnS | glutaminyl-tRNA synthetase |
| 589001 | + | TC**A** | TC**C** | Transversion | synonymous | - |  | 134 | 16 | 11% |  | 112 | 10 | 8% |  | 67 | 19 | 22% |  | FTT_0572 | - | proton-dependent oligopeptide transport (POT) family protein |
| 611902 | + | T**G**T | T**A**T | Transition | nonsynonymous | Cys 227 Tyr |  | 0 | 217 | 100% |  | 0 | 203 | 100% |  | 0 | 294 | 100% |  | FTT_0592 | cynT | carbonic anhydrase |
| 760327 | + | **G**GG | **A**GG | Transition | nonsynonymous | Gly 484 Arg |  | 0 | 329 | 100% |  | 0 | 269 | 100% |  | 0 | 362 | 100% |  | FTT_0738 | yjeF | carbohydrate kinase family protein (YjeF-related protein) |
| 801259 | - | TG**G** | TG**T** | Transversion | nonsynonymous | Trp 3 Cys |  | 120 | 1 | 1% |  | 146 | 4 | 3% |  | 55 | 14 | 20% |  | FTT_0781c | - | hypothetical protein |
| 822475 | + | G**C**A | G**A**A | Transversion | nonsynonymous | Ala 169 Glu |  | 248 | 25 | 9% |  | 220 | 35 | 14% |  | 225 | 65 | 22% |  | FTT_0803 | ans | asparaginase |
| 917551 | + | **A**TT | **C**TT | Transversion | nonsynonymous | Ile 238 Leu |  | 243 | 114 | 32% |  | 153 | 116 | 43% |  | 177 | 136 | 43% |  | FTT_0908 | parB | chromosome partition protein B |
| 973678 | + | TA**T** | TA**G** | Transversion | nonsense mutation | Tyr 27 Stop |  | 167 | 67 | 29% |  | 185 | 68 | 27% |  | 236 | 67 | 22% |  | FTT_0962 | - | ThiJ/PfpI family protein |
| 1074493 | - | CT**T** | CT**G** | Transversion | synonymous | - |  | 118 | 25 | 17% |  | 120 | 30 | 20% |  | 82 | 31 | 27% |  | FTT_1066c | - | hypothetical protein |
| 1155210 | + | **C**AG | **A**AG | Transversion | nonsynonymous | Gln 64 Lys |  | 118 | 23 | 16% |  | 102 | 8 | 7% |  | 44 | 13 | 23% |  | FTT_1143 | - | hypothetical protein |
| 1291591 | - | CC**T** | CC**G** | Transversion | synonymous | - |  | 82 | 8 | 9% |  | 93 | 14 | 13% |  | 25 | 7 | 22% |  | FTT_1269c | dnaK | molecular chaperone DnaK |
| 1327663 | - | A**A**G | A**C**G | Transversion | nonsynonymous | Lys 16 Thr |  | 266 | 33 | 11% |  | 235 | 26 | 10% |  | 153 | 40 | 21% |  | FTT_1304c | murB | UDP-N-acetylenolpyruvoylglucosamine reductase |
| 1339405 | - | **A**TT | **C**TT | Transversion | nonsynonymous | Ile 141 Leu |  | 226 | 91 | 29% |  | 170 | 60 | 26% |  | 191 | 14 | 7% |  | FTT_1313c | greA | transcriptional elongation factor |
| 1417127 | + | A**A**T | A**C**T | Transversion | nonsynonymous | Asn 9 Thr |  | 180 | 46 | 20% |  | 157 | 84 | 35% |  | 200 | 105 | 34% |  | FTT_1370 | - | hypothetical protein |
| 1482488 | - | T**C**A | T**T**A | Transition | nonsynonymous | Ser 182 Leu |  | 0 | 208 | 100% |  | 0 | 215 | 100% |  | 0 | 172 | 100% |  | FTT_1435c | yagD | ABC transporter, ATP-binding protein |
| 1634121 | - | G**G**T | G**A**T | Transition | nonsynonymous | Gly 659 Asp |  | 0 | 252 | 100% |  | 0 | 223 | 100% |  | 0 | 224 | 100% |  | FTT_1573c | - | outer membrane protein |

*^a^* Numbers indicated the genomic position of the *Francisella tularensis* subsp. tularensis SCHU S4 strains. (GenBank Accesion number, NC_006570.2)

*^b^* Bold and underlined nucleotides indicate the mutation position.

*^c^* Read depth indicates the number of short-read sequences mapped to each mutation position.
