# Supplementary material for: Role of Pathogenicity Determinant Protein C (PdpC) in Determining the Virulence of the Francisella tularensis Subspecies tularensis SCHU
Source: PLoS One. 2014 Feb 18;9(2):e89075. doi: 10.1371/journal.pone.0089075 (PMC3928404; doi:10.1371/journal.pone.0089075)
Supplement: Table S4 — Insertion and deletion position and mix population rate among P0, P5 and P9 based on SCHU S4 genome sequence. (DOCX) [file pone.0089075.s005.docx]

**Table S4. Insertion and deletion position and mix population rate among P0, P5 and P9 based on SCHU S4 genome sequence.**

| Genomic postion*^a^* | |  | Strand of described nucleotide |  | Detected mutation*^b^* | | | | | | | | | |  | Read depth*^c^* | | | | | | | |  | Genetic information on variation | | |
| --- | --- | --- | --- | --- | --- | --- | --- | --- | --- | --- | --- | --- | --- | --- | --- | --- | --- | --- | --- | --- | --- | --- | --- | --- | --- | --- | --- |
| insertion | deletion |  |  |  |  |  | P0 | |  | P5 | |  | P9 | |  | P0 | |  | P5 | |  | P9 | |  | locus tag | gene name | product |
|  |  |  |  |  | SCHU S4 |  | Identical sequence with SCHU S4 | Variant sequence |  | Identical sequence with SCHU S4 | Variant sequence |  | Identical sequence with SCHU S4 | Variant sequence |  | Identical sequence with SCHU S4 | Variant sequence |  | Identical sequence with SCHU S4 | Variant sequence |  | Identical sequence with SCHU S4 | Variant sequence |  |  |  |  |
|  | 635245 - 635239 |  | - |  | **A**AAAAAA |  | - | AAAAAA |  | - | AAAAAA |  | - | AAAAAA |  | 0 | 263 |  | 0 | 251 |  | 0 | 299 |  | FTT_0615c | - | metal ion transporter protein |
|  | 796219 - 796214 |  | - |  | **A**AAAAA |  | - | AAAAA |  | - | AAAAA |  | - | AAAAA |  | 0 | 138 |  | 0 | 142 |  | 0 | 185 |  | FTT_0776c | rnd | ribonuclease D |
|  | 1394871 - 1394879 or  1788206 - 1788214 |  | + |  | **A**AAAAAAAA |  | - | AAAAAAAA |  | - | AAAAAAAA |  | AAAAAAAAA | AAAAAAAA |  | 0 | 401 |  | 0 | 388 |  | **304** | **218** |  | FTT_1354 or FTT_1709 | pdpC1 or pdpC2 | hypothetical protein |
| 1540417 - 1540425 |  |  | + |  | AAAAAAAAA |  | - | **A**AAAAAAAAA  And  **AA**AAAAAAAAA |  | AAAAAAAAA | **A**AAAAAAAAA |  | AAAAAAAAA | **A**AAAAAAAAA |  | 0 | 291 and 3 |  | 4 | 213 |  | 2 | 243 |  | Intergenic region (FTT_1486c - 1487) |  |  |
|  | 1759054 - 1759179 |  | + |  | CGTTTAGGAA**CTATTATATAGTTTTTAGAATAAAAAATTATTTTTTTAATTTTGTTTTCTTCAGCATGATACTTTATCAAACATTTAAGTCCTTGTGGCTTCTCACTAAGTGTCTT**GGGTAAAGAA |  | - | CGTTTAGGAAGGGTAAAGAA |  | - | CGTTTAGGAAGGGTAAAGAA |  | - | CGTTTAGGAAGGGTAAAGAA |  | 0 | 268 |  | 0 | 187 |  | 0 | 247 |  | Intergenic region (FTT_1689c - 1690) |  |  |
|  | 1828482 - 1828479 |  | - |  | **A**AAA |  | - | AAA |  | - | AAA |  | - | AAA |  | 0 | 253 |  | 0 | 186 |  | 0 | 214 |  | FTT_1738c | kdpB | potassium-transporting ATPase B chain |

*^a^*Numbers indicated the genomic position of the *Francisella tularensis* subsp. tularensis SCHU S4 strains. (GenBank Accesion number, NC_006570.2)

*^b^* Bold and underlined nucleotides indicate the mutation position.

*^c^* Read depth indicates the number of short-read sequences mapped to each mutation position.
